# The Adaptive Function of Masturbation in a Promiscuous African Ground Squirrel

**DOI:** 10.1371/journal.pone.0013060

**Published:** 2010-09-28

**Authors:** Jane M. Waterman

**Affiliations:** Department of Biology, University of Central Florida, Orlando, Florida, United States of America; University of Plymouth, United Kingdom

## Abstract

**Background:**

Studies of animal mating systems increasingly emphasize female multiple mating and cryptic sexual selection, particularly sperm competition. Males under intense sperm competition may manipulate sperm quantity and quality through masturbation, which could waste sperm and decrease fertility. I examined the factors influencing masturbation by male Cape ground squirrels (*Xerus inauris*) in light of a number of functional hypotheses.

**Methodology:**

Observational data on a marked population of squirrels were collected in east-central Namibia using scan and all-occurrences sampling.

**Findings:**

Masturbation was far more frequent on days of female oestrus and mostly occurred after copulation. Masturbation rates were higher in dominant males, which copulate more, than in subordinates and increased with number of mates a female accepts.

**Conclusions:**

These results suggest that masturbation in this species was not a response to sperm competition nor a sexual outlet by subordinates that did not copulate. Instead masturbation could function as a form of genital grooming. Female Cape ground squirrels mate with up to 10 males in a 3-hr oestrus, and by masturbating after copulation males could reduce the chance of infection. Sexually transmitted infections (STIs) can profoundly affect fertility, and their consequences for mating strategies need to be examined more fully.

## Introduction

Male reproductive success can be strongly affected by the quantity or quality of sperm produced, especially in promiscuous mating systems with high levels of sperm competition. Sperm competition, in which sperm of different males compete for fertilization of ova within the female's reproductive tract [1], has resulted in morphological adaptations such as larger testes and larger accessory glands [2], [3] and behavioural strategies such as mate guarding and repeated copulations within the same oestrus [4]. Males with high risk of sperm competition are selected to increase sperm viability, ejaculate volume, or sperm concentration, suggesting that high sperm numbers are important under intense sperm competition [3], [5]–[7]. Many animals modify the number or quality of sperm in response to increased sperm competition [8]–[12].

Sperm counts decrease with successive ejaculations in many species [5], [12]–[14], suggesting that the rate of sperm production or maturation may be limited [13], [15]–[17]. Thus behaviours that waste sperm would appear to be maladaptive. For example, masturbation (manual stimulation of the sex organs to ejaculation) removes sperm without any possibility of fertilization. Yet, masturbation has been observed in many primates, rodents, and other species [18], suggesting it may have some adaptive function.

Only a few hypotheses have been proposed to explain the occurrence of masturbation (Table 1). The sexual outlet hypothesis suggests that masturbation is not adaptive but is instead merely a by-product of selection for neuroendocrine mechanisms that lead to increased sexual arousal and performance in promiscuous groups [19]. Males that have not successfully copulated might masturbate to ejaculation as a sexual outlet, particularly in species where multiple mating has selected for high sexual arousal [19]. Alternatively, masturbation could be adaptive and function to remove old sperm from the reproductive tract to increase the proportion of competitive or fertile sperm in the next copulation [20], [21]. Under this hypothesis, increased sperm competition should lead to increased rates of masturbation [20].

**Table 1:** Why masturbate? Hypotheses, predictions, and results for Cape ground squirrels.

Hypothesis		Prediction	Supported
1. Sexual outlet			
		Masturbation is more frequent on oestrus days	Yes
		Non-copulating males masturbate more	No
		Low-ranking males masturbate more	No
2. Improve sperm quality			
		Masturbation is more frequent on oestrus days	Yes
		Masturbation increases with number of mates	Yes
		Males masturbate before copulating	No
3. Reallocate energy			
		Masturbation increases in dry season	No
		Masturbation increases with oestrus length	No
4. Advertise to potential mates			
		Masturbation is more frequent near potential mates	No
5. Advertise to rivals			
		Males masturbate after copulating	Yes
		Oestruses with higher rates of masturbation will be shorter	No
6. Genital grooming to reduce STI			
		Masturbation is more frequent on oestrus days	Yes
		Masturbation increases with number of mates	Yes
		Males masturbate after copulating	Yes

In addition to these two previously proposed hypotheses to explain the evolution of this behaviour, I suggest other adaptive explanations that have not been addressed in the literature. Each of these hypotheses generates several testable predictions (Table 1). One possible function of masturbation could be to reallocate energy or water. Mammalian ejaculates contain substances that have energetic value that assist in sperm mobility [22] and males may use this energy, especially during energetically expensive mate searching. Masturbation also could be a form of advertising or signalling to future mates or competitors. Males may advertise their high quality to potential mates, signalling that they have high quantities of sperm and can afford to waste some. If masturbation is a signal of a successful copulation, then it could signal potential mates that they were the preferred mate of other females in order to encourage them to copy that mate choice [23], or to competitors that they have already copulated with the female so other males may cease searching (assumes a first male advantage in fertilisation). Lastly, masturbation may be a form of genital grooming, where males use the accessory gland fluids to cleanse the reproductive tract and reduce the transmission of sexually transmitted infections (STI).

I examined the occurrence and frequency of masturbation in a social, highly promiscuous rodent, the Cape ground squirrel (*Xerus inauris*), which inhabits the arid regions of southern Africa. Many traits of Cape ground squirrels suggest intense sperm competition is important in male mating success, including a large scrotum (20% of head-body length), a long penis (>40% head-body length), repeated matings, and high operational sex ratios (11∶1 males:female) during the short 3-h oestrus [24], [25]. During observations of free-ranging animals, I also observed males masturbating. This paper examines the occurrence of masturbation in light of the hypotheses and predictions in Table 1.

## Materials and Methods

### Ethics Statement

All squirrels were trapped and handled according to protocols approved by the Animal Care and Use Committee of the University of Central Florida (#07-43W).

### Biology of the study animal

Female Cape ground squirrels live in social groups characterized by female philopatry and male-biased dispersal, with 1–3 adults and up to 9 related sub-adults of either sex per group [26]. A single group inhabits a burrow cluster, and group members share sleeping burrows and a common feeding range [26]. Females may breed throughout the year and oestrus is highly asynchronous between and among social groups (it is extremely rare to have more than one female in oestrus on the same day). Each female can breed up to 4 times a year, and oestrus lasts an average of 3 h [24], [27]. Oestrus can be determined by the degree of female vulval swelling and by the behaviour of males (e.g., on days of oestrus, males sniffed, chased and copulated with the oestrous female [24], [27], [28]).

Adult males are scrotal throughout the year and can easily be distinguished from sub-adult males that are non-scrotal [26]. Adult males form all-male bands (up to 19 males) that are independent of female groups and persist throughout the year. Males within the band form sub-bands and the composition and size of these sub-bands changes daily [26]. Males usually sleep in vacant burrow clusters and share a large, undefended feeding range that overlaps with several female social groups, and they regularly travel throughout this range assessing the reproductive status of females [26], [29]. Aggression amongst males is extremely rare [26] but males form and maintain stable, linear dominance hierarchies determined by non-aggressive displacements (after an approach, one male jumps back from another), with older males (>2 years of age) being the most dominant [24], [26]. When a female comes into oestrus, up to 18 males have been observed to congregate on the burrow cluster area and begin searching for the female (searching consists of running through the area, briefly approaching other squirrels, and entering burrows where the oestrous female may have entered). This continual searching continues until the conclusion of oestrus [24]. Copulatory success is highly influenced by dominance rank; the most-dominant males find more oestrous females and gain first access during oestrus [24]. The first male to copulate most likely sires the offspring, and there is no evidence of mate guarding or copulatory plugs in this population. The operational sex ratio (ratio of receptive or adult males to receptive females during an oestrus) is not related to the number of mates accepted by the female or to the occurrence of repeated copulations [24]. Repeated copulations (number of copulations per male) in an oestrus increase with the number of mates with which a female copulates [24].

### Data collection

Field data were collected in east-central Namibia from 1989 to 1991 (23°25′S, 18°00′E). Temperatures range from −5 to 42°C in this region, but lowest temperatures are during the austral winter (June to August). Most annual rainfall occurs between November and April, and outside this period there is little to no rainfall (not enough to stimulate plant growth [30]). Thus, the wet season for this site was defined as the 6-month period from November to April and the dry season was the 6-month period from May to October [31].

All squirrels were trapped and marked for identification at a distance [24]. I trapped and marked all squirrels in 12 burrow clusters (12 female groups; 2 male bands) using Tomahawk (15×15×15 cm) and Havahart (21×21×90 cm) live traps baited with peanut butter and crushed corn. Traps were checked approximately every half-hour to 45 minutes. Individuals were marked with small freeze marks for permanent identification [32] and with hair dye (Rodol D, Lowenstein and Sons Inc., New York, NY; [33]) for identification at a distance. Squirrels were caught periodically to renew dye marks and assess reproductive condition. For details on trapping and marking, see [26], [27]. Male age was distinguished by fur condition in that males that are older than 2 years have a reduction of fur to the face area. The age of some males was also known from their emergence as juveniles or the date when they became scrotal (which occurs at 8 months [26], [27]).

I observed the squirrels for 2000 h with 10×50 binoculars from trees or a vehicle situated within 40 m of the perimeter of a burrow cluster. The identity, location, and activities of all squirrels were recorded using scan sampling at 5-min intervals [34]. I recorded interactions among males, masturbations, and male behaviours using all-occurrences sampling [24]. To determine the dominance ranks of males, I used the all-occurrence data of displacements, which occur when one individual approaches another (one individual moves directly up to another, within 10 cm) and the approach is followed by a ‘jumping back’ by one of the two males involved in the interaction [24], [26]. Dominance hierarchies were all linear (Landau's index of linearity >0.9) and transitive [24], [26], [35]. The most dominant male was assigned a rank of 1 and subordinates were assigned ranks that reflected the number of males dominating them [24], [35]. Locations of squirrels during observation were recorded using a grid marked with coloured flags or painted rocks placed at 10-m intervals within burrow clusters and at 20-m intervals in areas adjacent to the burrow cluster. I considered individuals to be near to each other if they were within 10 m of one another [26], [29]. I used focal-animal sampling of oestrous females to record all interactions with males, including successful copulations [24]. I observed 31 oestruses in their entirety (on 16 females) and recorded partial information on an additional 11 oestruses (4 additional females). An oral masturbation was recorded when a male sat with head lowered and an erect penis in his mouth, being stimulated with both mouth (fellatio) and forepaws (masturbation), while the lower torso moved forward and backwards in thrusting motions, finally culminating in an apparent ejaculation, after which the male appeared to consume the ejaculate. Because both the mouth and forepaws were used during this behaviour, I will use the term masturbation.

### Data analysis

For each male of reproductive age, I calculated the rate of masturbation as the total number of masturbations observed divided by the number of hours of observation for that male. To test the prediction that masturbation occurs more frequently on days of oestrus (Hypotheses 1, 2, and 6 in Table 1), I compared rates of masturbation on oestrous and non-oestrous days using a Wilcoxon signed-ranks test [36]. To test the prediction that non-copulating males masturbate more (Hypothesis 1), I calculated the % of oestruses where a male masturbated and copulated to the percent of oestruses where he masturbated and did not copulate and compared this with a Wilcoxon signed-ranks test. I used a Spearman's correlation of masturbation rates versus dominance rank to evaluate the prediction that low-ranking males would masturbate more (Hypothesis 1). Similarly, I tested if masturbations were influenced by the degree of sperm competition (Hypotheses 2, 6) by using a Spearman's correlation of the rate of masturbation and the number of mates accepted in an oestrus. To evaluate the timing of masturbation in relation to copulation (Hypotheses 2, 5, 6), I compared the percent of a successfully mated male's masturbations that occurred before, between or after copulation using a Friedman's nonparametric two way ANOVA. I compared the rates of masturbation in the dry and wet seasons using a Wilcoxon signed-ranks test to test the prediction that there was seasonality in masturbation (Hypothesis 3). I used Spearman's correlations to test if masturbation was related to the length of an oestrus (Hypotheses 3, 5). To evaluate if males masturbated more near potential mates (Hypothesis 4), I calculated the percent of all masturbations that occurred on a day of oestrus near a potential mate (adult female) excluding the breeding female.

I tested data for normality (Shapiro-Wilk test) and equal variance and used parametric tests for data that met assumptions; otherwise I used non-parametric statistics [36]. A 0.05 probability of a type I error was considered significant, and results are expressed as mean ±1 SE unless otherwise stated.

## Results

The majority of females mated more than once (90.3%, *N* = 31 oestruses of 16 females), copulating with an average of 4.3±0.45 males (range 1–10, *N* = 31). All 20 of the males observed masturbated to ejaculation and consumed the ejaculate. I observed 105 masturbations by these males in total.

**Figure 1:**
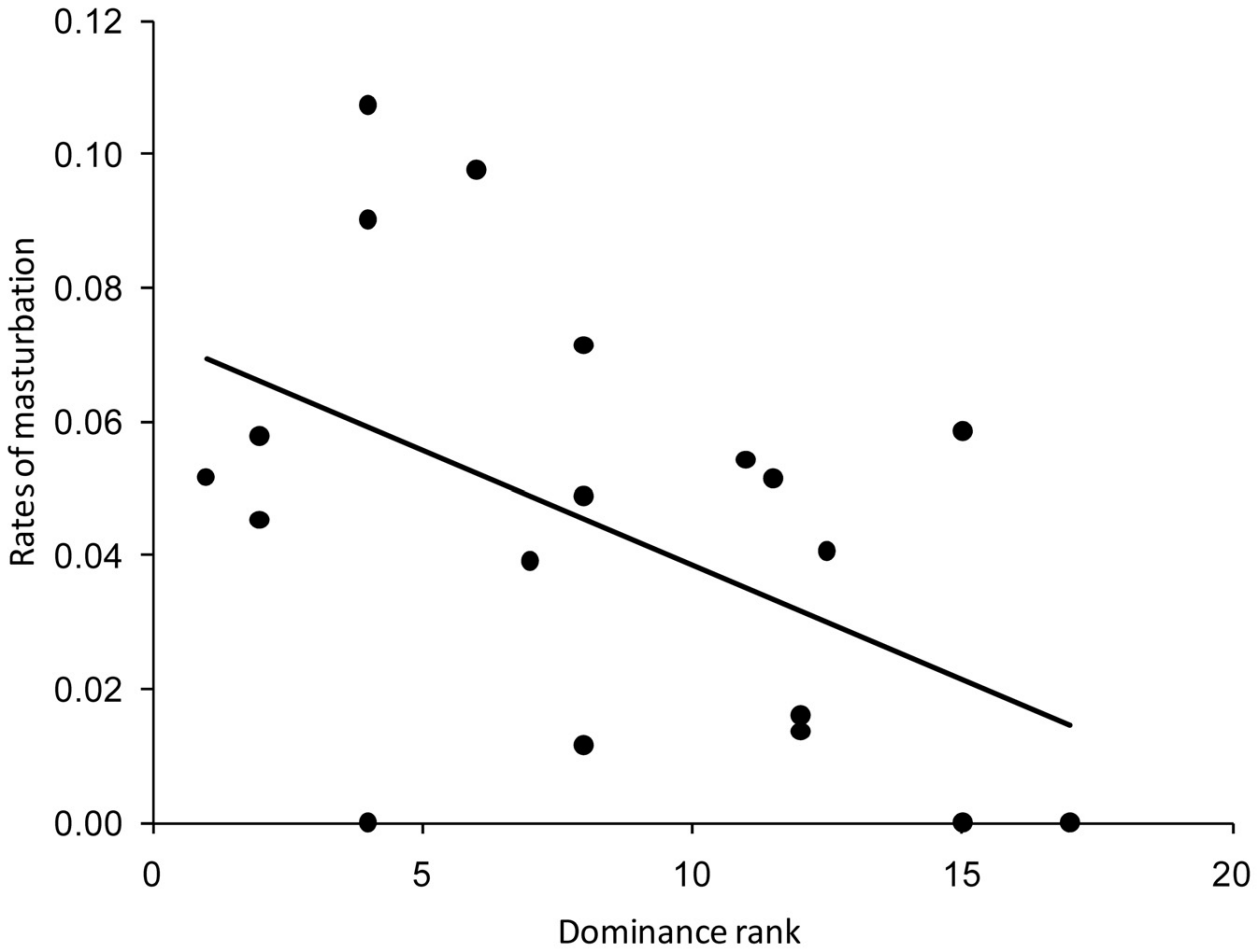
Rates of masturbation versus dominance rank. Each point reflects the masturbation (no./male/hr) of a single Cape ground squirrel during periods of female oestrus and his median rank. The most dominant male has a rank of 1.

**Figure 2:**
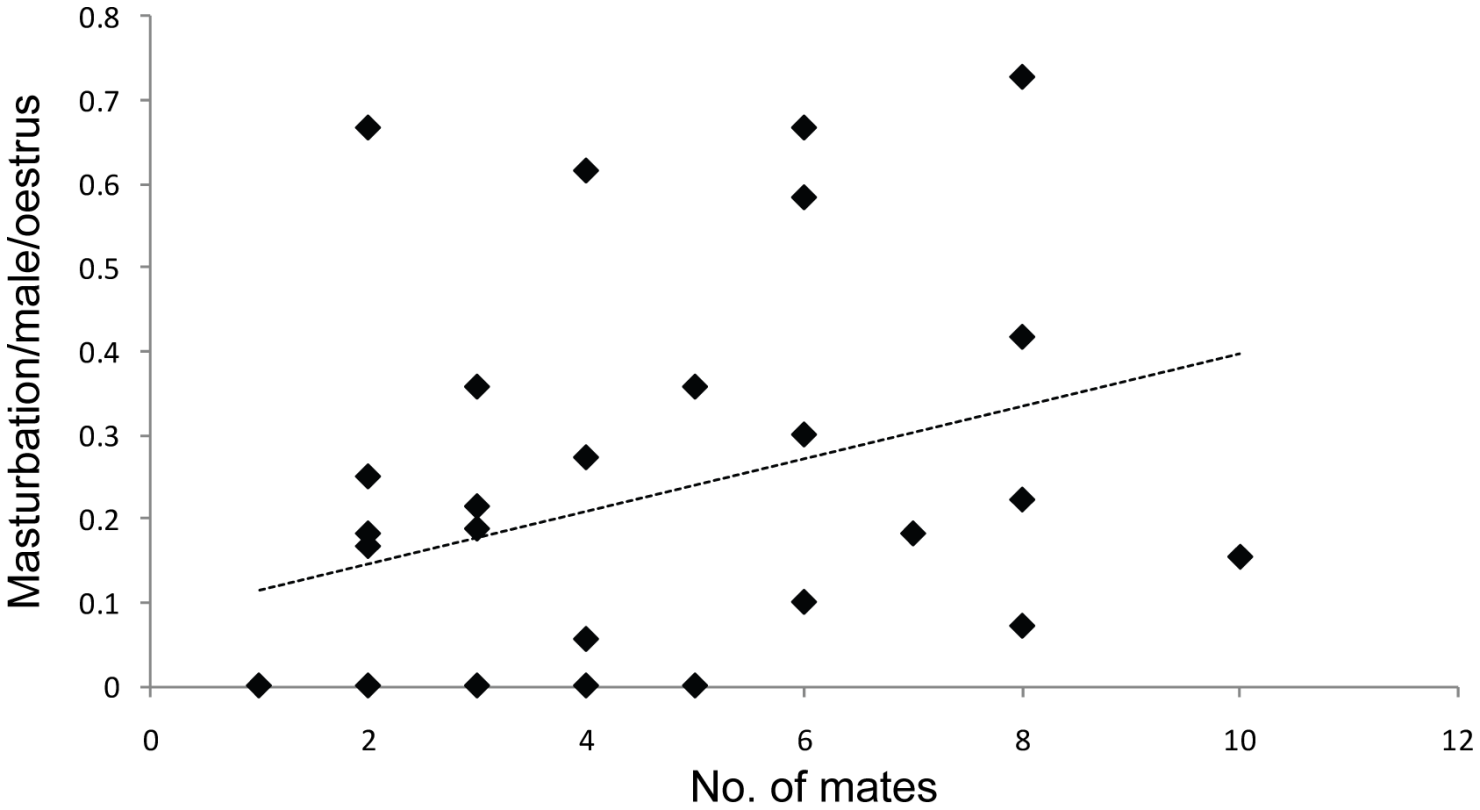
Rates of masturbation versus number of mates accepted. Masturbation (no./male/oestrus) by male Cape ground squirrels increased with the number of mates a female accepted. Each point reflects a single oestrus.

**Figure 3:**
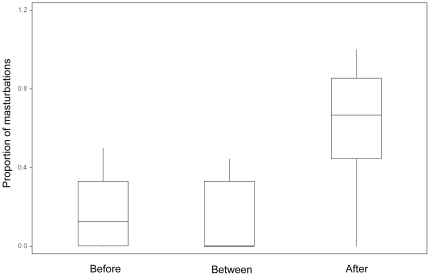
Box plot of the median and range of timing of masturbation by male Cape ground squirrels with respect to copulation.

Most masturbations (78%) were observed on days of female oestrus. Masturbation rates were much higher on days of oestrus (0.195±0.042/h) than on non-breeding days (0.025±0.007; *T* = 3.56, *P* = 0.0004, *N* = 20 males). Furthermore, males were more likely to masturbate during oestruses where they copulated (40.0±7.3% of successful oestruses) than at oestruses where they did not copulate (16.0±5.8% of unsuccessful oestruses; *T* = 2.48, *P* = 0.013, *N* = 20 males). Rates of masturbation were related to dominance rank, with the most dominant males masturbating more than subordinates (*r*_s_ = −0.48, *P* = 0.032, *N* = 20 males; Fig. 1). Mean number of masturbations per oestrus increased with the number of males a female mated with (*r*_s_ = 0.41, *P* = 0.023, *N* = 31 oestruses of 16 females; Fig. 2) but not with the number of males present at the oestrus (no. adult males per receptive female; *r*_s_ = 0.22, *P* = 0.237, *N* = 31). The likelihood of masturbation occurring was not affected by the order (first mate, second mate etc.) in which a male copulated (Likelihood Ratio Test, χ^2^_18_ = 13.9, P = 0.73). For 17 males that copulated at least once and masturbated during female oestrus, I calculated the percent of his masturbations that occurred before the first copulation, between copulations, and after the last copulation. Significantly more masturbations occurred after copulating than either before or between copulations (Fig. 3; Friedman test, χ^2^ = 12.9, *P* = 0.0015, N = 17 males that copulated and masturbated; a Friedman's post-hoc multiple comparisons test [37] indicated the number of after-copulation masturbations differed significantly (P<0.05) from both before and between-copulation masturbations).

Masturbation was not seasonal; masturbation occurred on 12.9% of 215 observation days in the dry season and 14.8% of 171 observation days in the wet season (Pearson's χ^2^ = 0.32, *P* = 0.57). Similarly, masturbation during oestrus did not differ seasonally, occurring during 56.0% of 25 of dry season oestruses and 52.9% of 17 wet season oestruses (Pearson's χ^2^ = 0.01, *P* = 0.92). Rates of masturbation in the dry season (0.06±0.013/h) did not differ from rates in the wet season (0.11±0.04/h; Wilcoxon signed-ranks test, *T* = 0.83, *P* = 0.41, *N* = 20 males) nor did they differ seasonally during oestrus (averaged 0.22±0.050 in the wet season and 0.19±0.085 in the dry season; *T* = 0.76, *P* = 0.45, *N* = 20 males). Length of oestrus (in hrs) did not affect rates of masturbation (Spearman rank correlation, *r*_s_ = 0.24, *P* = 0.19, *N* = 31). Only 17% of all masturbations (20 of 116) occurred when a potential mate was nearby (within 10 m).

## Discussion

Rates of masturbation in Cape ground squirrels were higher on days of female oestrus, increased with the number of mates accepted by the female, and usually occurred after a male had copulated with a female. Such behaviours that appear to waste huge numbers of sperm are a evolutionary puzzle because although individual sperm may be cheap, the actual ejaculate could be costly to produce [15], [38]. Furthermore, a delay in producing new sperm following ejaculation could impair a male's ability to fertilize a female, particularly in species like Cape ground squirrels, where males respond to increased sperm competition by repeatedly mating with the female [24].

The sexual outlet hypothesis predicts that males who were unsuccessful in copulating (particularly subordinate males) would be more likely to masturbate ([19]; Table 1). The increase in masturbation by Cape ground squirrels on days of oestrus is consistent with this hypothesis, but this hypothesis also predicts that males should masturbate less when they successfully copulate and rates of masturbation should decrease with dominance rank, since high ranking males have the highest copulatory success [24]. However, Cape ground squirrel males that copulated were more likely to masturbate than unsuccessful males and rates of masturbation increased with dominance rank.

Under the sperm quality hypothesis, increased sperm competition should lead to increased rates of masturbation [20]. In humans, masturbation increases sperm quality (by promoting younger sperm) without affecting sperm numbers in the female reproductive tract, suggesting this behaviour has arisen as a consequence of sperm competition [20]. This hypothesis predicts that masturbation should occur when females are in oestrus, and rates of masturbation should increase when sperm competition is higher. Both of these predictions were supported in this study. However, males masturbated more after copulating with the female than before, which suggests masturbation does not function to increase sperm quality.

Another possible function of masturbation could be to reallocate energy or water. Cape ground squirrel males appear to spend a lot of energy during oestrus searching for females, attempting to copulate, and disrupting copulation attempts of competitors [24], and the low resource availability during the dry season could constrain the energy budget of males. If Cape ground squirrels were to masturbate for energy or water during the intense mate searching on days of oestrus, rates of masturbation should be higher in the dry season and during longer oestruses. However there was no seasonal difference in masturbation, even when I looked at only days of oestrus, and there was no relationship between the length of oestrus and rates of masturbation.

Masturbation could also function as a form of advertising or sexual displays to future mates. However, interactions between males and potential mates (other than the female in oestrus) are rare on days of oestrus, as other members of the social group usually leave the burrow area upon first emergence and any interactions with males and non-oestrus females are agonistic [27], [29]. Males could be using masturbation as a signal to competitors that they have already copulated with the female so other males may cease searching. This hypothesis predicts that the occurrence of masturbation should shorten the length of time males continue to seek the female in species with a first male advantage in fertilisation. Evidence supports a first male advantage in Cape ground squirrels [24], but there was no relationship between rates of masturbation and the length of oestrus, suggesting males continue to mate with females regardless of a masturbation display. However it is possible that males are observing successful males masturbating. Although it is unlikely that masturbation functions as a signal, this hypothesis cannot be excluded without more testing.

Another possible explanation is that masturbation functions to remove potential infections transferred from a female that has previously mated – a form of genital grooming. Sexually transmitted infections (STIs) can have profound effects on fitness, even if there are no apparent symptoms [39]. Just having an immune response to infection can affect human male fertility, including ejaculate volume, sperm concentration, sperm mobility, and sperm morphology [39]. This hypothesis predicts that masturbation should occur on a day of oestrus, after successfully copulating, and should increase with the number of mates a female accepts (Table 1). All of these predictions were supported by the masturbation data of Cape ground squirrels.

Postcopulatory behavioural mechanisms to reduce STIs have mostly focused on genital grooming or urination [40]. Genital grooming following copulation is common in rats [41] and in primates [40] and reduced or prevented infection by STIs in experiments on rats [41], [42], probably because of the anti-bacterial and anti-viral properties of saliva [42]. Reduction of STIs has also been suggested as an explanation of fellatio in bats [43]. Like other rodents, both sexes of Cape ground squirrels will genital groom after mating [24] and they may benefit from the antibacterial properties of saliva. Postcopulatory urination may be used by humans, especially males, to avoid infection, as the urethra is the primary site of infection of many STIs [44]. As a desert-adapted species, however, Cape ground squirrels produce very concentrated urine and rarely urinate [45], [46] and I never observed urinating after copulation. For organisms that rarely urinate, masturbation may serve a similar function to postcopulatory urination, as a more thorough mechanism to clean vital reproductive tracts after mating than just external genital grooming. Consuming the ejaculate may prevent moisture loss.

Furthermore, the antibacterial nature of accessory gland secretions has been well-documented in humans and other mammals [47], [48], and species under more intense sperm competition have larger accessory glands [3]. These larger glands are thought to aid in forming copulatory plugs to block fertilization attempts by subsequent males [3], but since promiscuity increases potential transmission of infection, these glands also could be selected for their antibacterial benefits.

Of the 6 hypotheses examined to explain the function of masturbation in Cape ground squirrels, four were unsupported by the data. Masturbation as a mechanism to reduce STIs had the most support. Multiple mating by females as well as males is much more common than previously recognized [4] and can positively affect fitness, but the risks of STIs are not as well-documented in wildlife. Exploring masturbation as a mechanism to reduce STIs in males may explain some of the masturbation patterns seen in multimale-multifemale primate groups [19] and even humans [20].
